# The effect of two different health messages on physical activity levels and health in sedentary overweight, middle-aged women

**DOI:** 10.1186/1471-2458-11-204

**Published:** 2011-03-31

**Authors:** Sebely Pal, Cheryl Cheng, Suleen Ho

**Affiliations:** 1 School of Public Health; Curtin Health Innovation Research Institute; ATN Centre for Metabolic Fitness; Curtin University of Technology, Perth, Western Australia

## Abstract

**Background:**

Most public health guidelines recommend that adults need to participate in 30 minutes of moderate intensity physical activity on most days of the week to maintain good health. Achieving the recommended 30 minutes of exercise a day can be difficult in middle aged, overweight women. This 12 week study evaluated whether a 10,000 steps per day message was more effective than a 30 minutes a day message in increasing physical activity in low active, overweight women.

**Methods:**

Thirty participants were randomized into 2 groups: Group 1 was asked to undertake 30 minutes of walking/day, whereas Group 2 was asked to accumulate 10,000 steps/day using their pedometers.

**Results:**

Results showed that there were no changes in anthropometric and blood pressure measures between or within groups. However, the 10,000 step and the 30 minutes groups' daily average number of steps/day were significantly higher than baseline at week 6 (p = 0.038 and p = 0.039 respectively) and at week 12 (p = 0.028 and p = 0.038 respectively). At week 12, the 10,000 steps group were taking an average of 4616 steps per day more (43% increase) than at baseline and the 30 minutes group were taking an average of 2761 steps per day more (35% increase) than at baseline. There was a significant difference in the number of steps with the 10,000 steps group versus 30 minutes group at 12 weeks (p = 0.045).

**Conclusions:**

This study found that low active, overweight women undertook significantly more physical activity when they had a daily 10,000 step goal using a pedometer, than when they were asked to achieve 30 minutes of walking/day. Therefore we suggest that a public health recommendation of "10,000 steps/day", rather than the "30 min/day" could be applied to promote increased physical activity in sedentary middle aged women.

## Background

It is estimated that there are 1.6 billion adults worldwide who are overweight and at least 400 million of them obese [1]. The increased consumption of convenience foods and increased sedentary activity from usage of technology such as the television (TV), computers and cars are examples of environmental factors playing a detrimental role in this obesity epidemic. The World Health Organization [1] estimate that 1.9 million deaths are attributable to physical inactivity. Numerous studies have found many health benefits of physical activity such as improvements in psychological well-being and also a decrease in the risk factors associated with chronic diseases such as diabetes, hypertension, cardiovascular disease (CVD) and obesity [2-7].

To maintain good health and reduce the risk of chronic disease, most public health guidelines recommend that adults need to participate in 30 minutes of moderate intensity physical activity on most days of the week [8-11]. Achieving such a target can be difficult in sedentary groups and especially in overweight/obese populations, therefore inventing simple, easy to implement methods which help to provide environmental cues and motivation to increase physical activity should be investigated. Previous studies have shown that the daily use of pedometers, a quantifiable walking measure, results in significant increases in physical activity [12]. The feedback received from pedometer step counts encourages behavior change as it raises awareness about current walking behaviors [13], and through self monitoring can motivate a person to increase their physical activity [13,14]. A meta-analysis of 26 randomised controlled trials (RCTs) and observational studies involving the daily use of pedometers in adults has reported significant increases in the number of steps walked per day [12]. A study by Hultquist et al. [15] compared the number of steps accumulated by two groups of women instructed to either walk 10,000 steps per day using pedometers or instructed to walk 30 minutes per day. The 10,000 steps group accumulated significantly more steps (p < 0.005) than the 30 minutes group, indicating that pedometer use was more effective than the 30 minutes message in increasing walking activity in this group over 4 weeks. Thus, they suggest that public health guidelines should change their 30 minutes/day message to a 10,000 steps per day to promote physical activity in the adult population. In a follow-up study by the same group of investigators, they observed that daily steps were still significantly greater after 12 months compared to baseline, however there were no longer any significant differences between the 30 minutes and the 10,000 steps groups at any time point after the initial 4 week intervention period [16]. No other studies have further compared the effectiveness of these two messages for a longer period greater than 4 weeks. The intervention of 4 weeks may not be long enough to observe the true effect of these 2 messages after the initial novelty of wearing a pedometer has worn off. Further scientific evidence is required if modifications to current physical activity public health messages are to be made.

If sedentary individuals or those who undertake low activity have on average 5000-7499 steps per day [17] then the addition of the recommended 30 minutes of walking per day (equating to 3500 steps) would be close to 10,000 steps per day. Therefore the aim of this study was to evaluate whether a 10,000 steps per day message or the 30 minutes per day message was more effective in bringing about significant increases in physical activity in overweight and obese women over a three month period. We hypothesized that the former message would be more effective in promoting physical activity in sedentary, overweight women. In this study middle-aged women were targeted as > 50% of this group are overweight and therefore at high risk for developing cardiovascular disease, diabetes and hypertension[18]. Women are specifically targeted in this pilot study as they generally have a large input into their families. Thus, any lifestyle changes have the potential to flow on and affect their family members (spouses, children).

## Methods

Overweight and obese low active, middle aged (35-55 years) women with a body mass index (BMI) between 25-35 kg/m^2 ^were recruited from local community newspapers in Perth, Western Australia. Interested participants were screened and those who met the selection criteria attended a group orientation session where details of the study were explained. Exclusion criteria included current chronic medical and psychological disease, major systemic illness, renal failure, pregnancy, lactation or planning to become pregnant, smoking, hypothyroidism, diabetes mellitus, pre-existing heart conditions or gastrointestinal surgery and greater than two hours of moderate intensity physical activity per week. The Ethics Committee of Curtin University reviewed and approved all procedures and informed, written consent was obtained from each participant prior to participating in the study. Trial registration: ACTRN12609000176268.

Seventy eight women were screened. Thirty four women met the inclusion criteria and attending the orientation session. Two women dropped out of the study before commencement due to work commitments. Thirty two overweight/obese women were then randomized into two groups: Group 1 (30 minutes group) - with a goal to undertake at least 30 minutes per day of walking on top of their baseline activity, or Group 2 (10,000 steps group) - with a goal to accumulate at least 10,000 steps per day, over a twelve week period. The 30 minutes group wore a sealed pedometer for twelve weeks and therefore had no knowledge of their record of steps/day. The pedometer was sealed with tape so that the number of steps/day was concealed and this seal was only broken once a week by the participant so that the total weekly steps could be recorded on a calendar provided. On a weekly basis they were asked to reset the pedometer to zero and re-seal the pedometer. Participants acknowledged that they understood the instructions and would comply with them by signing a form to confirm this. The 10,000 steps group wore an unsealed pedometer which they were able to open and view the number of steps accumulated throughout the day. Participants in this group were instructed to record accumulated steps/day on a calendar and to reset the pedometer to zero each day. Subjects in both groups were asked to record each day when they wore their pedometers on a calendar supplied to monitor compliance.

Before the study commenced, all 32 participants wore a sealed Yamax Digi-Walker SW-200 pedometer to record the amount of steps they accumulated over a one week period, in order to collect baseline data. This pedometer has an overall mean absolute error of 3% [19]. Two studies have compared 13 pedometer models and found that Yamax Digiwalker SW-200 pedometer to be one of the most accurate and suitable for research [20,21]. The accuracy of the pedometer on each participant was checked by the means of a 20 (+/-2) step test at the outset. Participants wore the pedometer clipped to their clothing at the waist, centered over the foot.

At baseline, both groups were given the National Australian Physical Activity Guidelines [8] and information from these guidelines such as intensity, duration, prompts for exercise and relapse prevention were discussed with the participants. The 30 minutes group were specifically instructed to walk 30 minutes a day to comply with the physical activity guidelines and a 30 minutes/day walking goal was set. This could be achieved in a 30 minute block or in shorter bouts of walking of 10-15 minutes. The 10,000 steps group were instructed to reach a daily step goal of 10,000 steps per day. At baseline, participants from both groups were encouraged to initially set small achievable goals and then to gradually increase the walking goal each week to at least 30 minutes/day (30 minutes group) or 10,000 steps (10,000 steps group). Physical activity was assessed at baseline and at 12 weeks using the short-form International Physical Activity Questionnaire (IPAQ), which provides information on the time spent walking, in moderate physical activity, in vigorous physical activity and total physical activity (MET·min/wk) (MET = Metabolic Equivalent Task) during a usual week. This version of the IPAQ has been found to be valid and reliable [22].

Participants were asked to maintain their usual dietary intake throughout the 12 weeks of the study. Dietary intake was monitored through the completion of 3-day food diaries, including two week days and one weekend day, at the beginning and end of the study. Energy and macronutrient intakes from the participants' combined food records were calculated using Food Works (Version 3 Xyris Software, 2002) based on data from the AUSNUT database.

### Physical examination

At baseline, 6 and 12 weeks the participants attended at Curtin University for a brief physical examination, including blood pressure, weight, waist and hip measurements. Weight measurements were taken using Tanita scales (UM-018 Digital Scales, Tanita Corporation, Tokyo, Japan), with participants dressed in light clothing without shoes. Height measurements were taken using a mechanical stadiometer (Surgical and Medical Products, Hills, Australia. BMI (kg/m^2^) was calculated from weight and height measurements. Waist (at umbilicus) and hip circumference were measured from which Waist to Hip ratio (WHR) was calculated. Body composition was also measured using the RJL Systems BIA - 101 Body Composition Analyzer (USA) following the manufacturer's instructions. These standardized protocols have been established in our laboratory.

### Statistical analysis

Statistical analysis was conducted using SPSS 17 for Windows (SPSS Inc., Chicago, IL). Data are expressed as mean (SEM) and assessed for normality. Differences between groups were analysed using one-way analysis of variance. Data was analyzed further by post-hoc analysis using the Least Square Differences (LSD) method to identify specific differences. Statistical significance was considered at p < 0.05. Sample size calculation was based on a predicted change of 20% in steps/day between 30 minutes and 10,000 step groups as suggested in previous studies [12,15,23]. Assuming a standard deviation of 10%, a sample size of 12 participants per group provides sufficient power (80%) to detect changes at the 5% significance level. A total of 32 participants were recruited to ensure adequate numbers in the event of participants choosing to withdraw from the study.

## Results

Thirty two participants (16 participants/group) commenced the study, however four participants withdrew due to personal issues (travel and illness). Results were reported on the remaining 28 participants, with 13 participants in the 10,000 steps group and 15 participants in the 30 minutes group. Baseline characteristics of participants, such as height, weight did not significantly differ between the groups (Table 1). The average age was 41.38 ± 2.72 years and 45.27 ± 2.18 for the 10,000 steps group and 30 minutes group, respectively.

**Table 1:** Anthropometric and blood pressure measurements at baseline and week 12 for the 30 minutes and the 10,000 steps groups

Variable	Group			
	
	30 Minutes		10,000 steps	
	
	Baseline	Week 12	Baseline	Week 12
Body Mass Index (kg/m^2^)	29.70 ± 1.05	29.68 ± 1.11	28.90 ± 1.20	29.08 ± 1.03

% Body Fat (%)	39.03 ± 1.68	38.59 ± 2.00	38.34 ± 1.86	38.14 ± 1.62

Waist circumference (cm)	96.48 ± 2.07	95.38 ± 2.36	94.46 ± 2.34	93.45 ± 3.32

Systolic Blood Pressure (mm Hg)	115.26 ± 3.47	114.33 ± 3.66	112.20 ± 2.41	113.7 ± 3.34

Diastolic Blood Pressure (mm Hg)	64.06 ± 1.53	62.72 ± 1.81	63.22 ± 2.24	63.37 ± 2.27

Values are means ± SEM. Baseline characteristics were not significantly different between the two groups. There were no significant differences within groups or between groups in BMI, % body fat, waist circumference, or blood pressure at 12 weeks.

**Figure 1:**
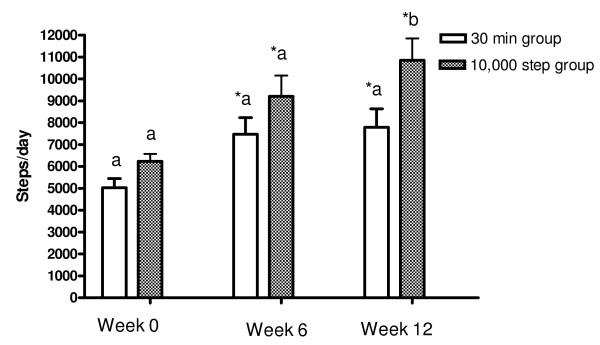
**Average number of daily steps taken at baseline, week 6 and week 12 by the 30 minute group and the 10,000 steps group**. The average steps/day were measured at baseline, week 6 and week 12 in both groups. Data are expressed as means ± SEM. Different letters above bar graphs indicate significance between groups at p < 0.05. * Significant difference (*P *< 0.05) within group (baseline versus 6 weeks and baseline versus 12 weeks).

Figure 1 shows the number of steps over 12 weeks for the 10,000 steps group and 30 minutes group. For the 10,000 steps group the daily average number of steps/day at weeks 6 and 12 was 9311 ± 954 and 10,847 ± 1006, respectively and was significantly higher than the baseline daily average of 6231.59 ± 342.34 steps/day (p = 0.038 and p = 0.028, respectively). At week 12, the 10,000 steps group were taking an average of 4616 steps per day more (43% increase) than at baseline. For the 30 minutes group, the daily average number of steps at weeks 6 (7472 ± 761 steps per day) and 12 (7786 ± 850 steps per day) were significantly higher than the baseline daily average of 5025 ± 416 steps per day (p = 0.039 and p = 0.038, respectively). At week 12, the 30 minutes group were taking an average of 2761 steps per day more (35% increase) than at baseline (5025.09 ± 416.08 steps/day). There was a significant difference in the number of steps with the 10,000 steps group versus the 30 minutes group at 12 weeks intervention (p = 0.045) but not at 6 weeks.

There were no significant differences within groups or between groups in waist, BMI, waist/hip ratio, % body fat or blood pressure at 12 weeks (Table 1). Table 2 shows that there were no significant differences between the groups in the macronutrient content of their diets at baseline or at 12 weeks.

**Table 2:** Nutritional analysis of food records 3 days prior to baseline and week 12 visits for the 30 minutes group and the 10,000 steps group

	Groups			
	
	30 minutes		10,000 steps	
	
NUTRIENT	Baseline	Week 12	Baseline	Week 12
Energy intake (kJ)	7825.46 ± 434.46	6749.92 ± 729.93	7456.45 ± 610.89	6724.36 ± 625.41
Carbohydrate (% of total energy)	46.85 ± 1.72	44.15 ± 2.93	44.23 ± 2.32	45.63 ± 3.21
Protein (% of total energy)	16.08 ± 0.66	19.38 ± 1.87	18.89 ± 1.56	18.45 ± 1.69
Total fat (% of total energy)	31.92 ± 1.77	33.85 ± 2.04	36.73 ± 1.76	34.45 ± 1.60

There were no significant differences in energy or macronutrient intake on the 3 days prior to baseline and week twelve visits of both groups. Values are means ± SEM.

Table 3 shows that there was a significant increase in time spent walking in the 10,000 steps group at 12 weeks compared to baseline (*P *= 0.034) and a non-significant increase in the 30 minutes group compared to baseline. There was no significant difference in time spent walking between groups at 12 weeks. Time doing moderate or vigorous physical activity was not significantly different between the 10,000 steps and the 30 minutes group at week 12. Although time spent undertaking moderate activity and total physical activity increased in the 10,000 steps group versus the 30 minutes group at 12 weeks, this was not significant.

**Table 3:** Physical activity data from the International Physical Activity Questionnaire (IPAQ) at baseline and 12 weeks for 10,000 steps and the 30 minutes group

	Groups				P value
	
Variable	30 minutes		10,000 steps		
	**Baseline**	**Week 12**	**Baseline**	**Week 12**	**Week 12**

Walking (MET·min/wk)	229.0 ± 117.5	445.4 ± 144.1	246.0 ± 120.0	648.3 ± 160.8*	0.110
Moderate activity (MET·min/wk)	183.6 ± 65.5	229.8 ± 62.9	172.5 ± 52.5	262.3 ± 71.5	0.330
Vigorous activity (MET·min/wk)	66.0 ± 26.3	80.3 ± 20.3	67.8 ± 17.0	93.5 ± 23.0	0.260
Total activity (MET·min/wk)	435.6 ±153.5	645 ± 165.3	445.7 ± 165.4	734 ± 215.5	0.230

Values are represented as mean ± SEM

There were no significant differences at baseline between groups.

*Significant difference (*P *< 0.05) within group (Baseline versus 12 weeks)

*P-values *represent the between group comparison of the 30 minutes group and the 10,000 steps group at week 12.

There were no significant differences between the 30 minutes group and the 10,000 step group at 12 weeks.

## Discussion

The purpose of this study was to compare the effect of a10,000 steps per day message and a 30 minutes per day message on physical activity levels in low active, overweight and obese women for 12 weeks. Our results show that the 10,000 step group and the 30 minutes group both had an increase in daily steps at 6 and 12 weeks compared with baseline. However, overweight and obese women accumulated significantly more total steps/day when instructed to walk 10,000 steps per day than when instructed to walk 30 minutes/per day after 12 weeks. In addition, those in the 10,000 steps group significantly increased their amount of walking at 12 weeks compared to baseline, while the 30 minutes group did not. Therefore a 10,000 step goal and immediate feedback from using a pedometer was more effective in increasing physical activity levels in low active overweight and obese women than the 30 minutes/day message.

Consistent with our study, the efficacy of having a step goal and using pedometers daily as a way of increasing walking activity has been demonstrated in a number of studies [12,15]. The four week study by Hultquist et al [15] comparing the number of steps accumulated by women instructed to walk 10,000 steps per day with those told to take a brisk 30-minute walk, concluded that women walk more when told to take 10,000 steps per day compared with those instructed to take a brisk 30-minute walk. This study by Hultquist et al [15] also gave their 30 minutes group a sealed pedometer that was only opened at the end of the week. After four weeks of intervention the 30 minutes group accumulated an average of 8270 ± 354 steps per day and the 10,000 steps group averaged 10,159 ± 292 steps per day. This was similar to our present study where the 10,000 steps group averaged 10,847 ± 1006 steps per day compared to the 30 minutes group who averaged 7786 ± 850 steps per day, after 12 weeks intervention. Another study conducted by our group examined whether the daily use of pedometers could increase physical activity and improve health outcomes in sedentary overweight and obese middle-aged women [24]. Participants were randomized into two groups: The control group wore a sealed pedometer, whilst the pedometer group were given a step goal of 10,000 steps/day, were able to use their pedometers for self-monitoring throughout the day and were asked to record the number of steps on a daily basis for 12 weeks. This previous study showed that the pedometer group significantly increased their steps/day, by 36%, at the end of the 12 weeks, whereas the control group's physical activity levels remained unchanged. This pilot study showed that the combination of having step goals and immediate feedback from using a pedometer was effective in increasing physical activity levels in sedentary overweight and obese women[24].

A meta-analysis by Bravata et al. [12] on pedometer use concluded that study participants who have a step goal and were able to record their steps daily, were able to significantly increase their physical activity levels compared to those who did not have a step goal. They found that those who used pedometers, had a step goal and recorded their steps, took an average of 2491 more steps a day compared to those who did not have a step goal. They also found that the step goals groups had an average increase of around 27% when compared to baseline levels. In our study, the 10,000 steps group had an increase of 43% compared to baseline levels whereas the 30 minutes group had an increase of 35% from baseline. It appears that participants in the 10,000 steps group were using their pedometer as a measurement tool as they had a quantitative measure and therefore could monitor their daily physical activity. If they had not achieved the 10,000 steps goal, this could motivate them to undertake more activity to reach the 10,000 steps goal [13,14]. In comparison, participants in the 30 minutes group were not able to view this information as they were not given a measurement tool for daily use to prompt them to meet their 30 minutes walking goal. Although they were given a daily walking goal of 30 minutes over their normal activity, such a goal was not sufficient to match the physical activity levels undertaken by the 10,000 steps group.

Public health guidelines currently recommend that around 30 minutes of moderate-intensity activity/day on most days of the week provides significant health benefits and can reduce the risk for a range of conditions [8,9,11]. Brisk walking for 30 minutes is equivalent to approximately 3500 steps per day [17,25,26]. Tudor-Locke et al [17] suggest that sedentary activity equates to < 5000 steps with 5000-7499 steps being classified as low active. Adding 30 minutes per day (3500 steps per day) to 5025-6231 steps, the baseline means of the two groups in our study, would be close to 10,000 steps per day. Both groups of participants were asked to accumulate approximately 10,000 steps per day. In our study, it appears that those in the 10,000 steps group were better able to comply with the physical activity guidelines with an extra 4616 steps steps/day above baseline steps per day than compared with the extra 2761 steps/day performed above baseline by the 30 minutes group. The 10,000 steps group had a 40% greater increase in steps/day than the 30 minutes group at 12 weeks. Given that 30 minutes of walking is estimated to be equal to approximately 3000-3500 steps, this suggest that the 30 minutes group, on average, did not comply to the 30 minutes/day walking intervention.

There were no significant differences in anthropometric measurements between the 10,000 steps group and 30 minutes group as also shown in other studies [27]. It is possible this could have been as a result of increased energy intake in conjunction with increased physical activity although this is not reflected in the dietary records (Table 2). It is well established however that under reporting of energy intake is widespread in overweight/obese populations [28,29] and could have occurred here. It is more likely that 10,000 steps/day or an extra 30 minutes/day on top of normal activity is insufficient to achieve weight loss [30]. Although public health guidelines recommend 30 minutes of moderate-intensity activity on most days to maintain good health [8,9,11], in order to achieve and maintain weight loss, the US Physical Activity Guidelines Advisory Committee [31] has now recommended that overweight/obese adults should achieve at least 60 minutes of physical activity on most days of the week. Saris et al [30] also suggest that moderate intensity exercise of around 60 - 90 minutes (an extra 6000-9000 steps/day), on most days would be necessary for any weight loss to occur. Tudor-Locke et al [17] suggest that brisk walking for 60 minutes equates to approximately 6,000 steps per day therefore overweight individuals would need to walk an extra 6000+ steps for weight loss. The accumulation of 4616 steps/day by the 10,000 steps group and extra 2761 steps/day accumulated by the 30 minutes group at 12 weeks compared to baseline steps/day may not have been sufficient to observe changes in anthropometric measures [32]. Increasing the steps to at least 12,000 per day for overweight adults would be required to achieve these new guidelines and to encourage weight loss although energy intake also would need to be monitored [31]. Further studies would be required to demonstrate whether pedometers used on a daily basis could encourage overweight individuals to achieve at least 60 minutes of activity on most days or at least 12,000 steps per day to achieve the new recommendations for this group [31]. Therefore, favorable changes in anthropometric measures may be achievable after 12 weeks with higher levels of physical activity.

A study limitation involved using a pedometer without a memory chip. A memory chip enables a daily step tally to be recorded which can indicate how many steps were taken on each day. This would have been useful in order to observe if the 30 minutes group, who despite not being able to see their daily steps, had higher step counts in the first 2-3 days in reaction to the study conditions, as has been shown previously [33]. Changing the type of pedometer for future studies with overweight and obese individuals may be appropriate as a piezo-electric pedometer has been shown to be more accurate for those with a higher BMI than a Yamax Digi-Walker [34].

All participants in the 30 minutes group were given instructions in the beginning of the study to only record weekly steps and reseal pedometers. They all signed a form to acknowledge that they understood these instructions and would abide by them. However, it was unknown whether there was potential non-compliance with resealing pedometers. It was also unclear whether the 30 minutes group observing their step counts on a weekly basis had an influence on their general activity levels.

Another study limitation involved limited information as to how participants undertook their physical activity. Although levels of activity were recorded, such as walking, moderate intensity and high intensity exercise [22], there was no indication as to whether the exercise was performed in one bout or in smaller bouts per day. It is recommended that exercise should be undertaken in 10 minute bouts and resistance exercise should also be incorporated twice a week [10,27] although this was not revealed to the study participants. Also, there can be variability between individuals as one person's exercise intensity can be different to another's even though the same number of steps are taken. The increase in the daily number of steps/day for both groups was mainly through walking activity as indicated by the IPAQ results. Walking has been shown to be the easiest way for individuals to increase physical activity levels and maintain them over the longer term [35]. Self paced walking is the most common physical activity reported by overweight and obese individuals and is preferred due to its low-impact nature and reduced risk of injury [8,36,37].

In this study, overweight and obese women accumulated more total steps/day when instructed to walk 10,000 steps per day than when instructed to walk 30 minutes/per day. It appears that having a step goal which can be monitored with the use of a pedometer, where progress can easily be tracked from a reference or baseline point, can act as a motivator [14] and enables self monitoring [13,14] which in turn leads to increased physical activity [7,38]. Setting a step goal in conjunction with the immediate feedback that the pedometer provides may be key motivational factors for increasing physical activity. Other studies have shown that immediate feedback from the pedometers enable participants to set realistic goals, act as an environmental cue, raise awareness of current walking behaviours [13], to motivate [39], to self-monitor [13,39], and to increase walking behaviours [7,38]. The present findings may be relevant to the wider sedentary overweight and obese population where using a pedometer and having a step goal may provide a promising strategy to increase physical activity.

## Conclusions

In conclusion, this study shows that a 30 minutes message and a 10,000 step message were both useful in promoting physical activity, however, the combination of having step goals and immediate feedback from using a pedometer in the 10,000 steps group was more effective in increasing physical activity levels in low active overweight and obese women. Therefore we suggest that a public health recommendation of "10,000 steps/day", rather than the "30 min/day" could be applied to promote increased physical activity in sedentary middle aged women.

## Authors' contributions

The authors' CC and SH coordinated the trial, data collection, statistical analysis and input into the manuscript. SP conceived and designed the study, wrote the manuscript, supervised the study and the statistical analysis. All authors read and approved the final manuscript

## Conflict of interest

The authors declare that they have no conflict of interest in relation to this manuscript.

## Pre-publication history

The pre-publication history for this paper can be accessed here:

http://www.biomedcentral.com/1471-2458/11/204/prepub
